# How to create PICO questions about diagnostic tests

**DOI:** 10.1136/bmjebm-2021-111676

**Published:** 2021-03-31

**Authors:** Hendrika J Luijendijk

**Affiliations:** Department of General Practice and Elderly Care Medicine, University of Groningen, University Medical Centre Groningen, Groningen, The Netherlands

**Keywords:** evidence-based practice, diagnosis, methods, women's health services

## Introduction

PICOs are popular in medical teaching.^1^ The acronym stands for Patient (participant, problem or population), Intervention or exposure, Control (comparator) intervention or exposure, and Outcome. It is a useful tool to teach students how to formulate questions about the effect of a treatment in a testable way, and search for answers in the medical literature. A PICO is also helpful when designing and reporting a systematic review.

Less obvious, but another strength of a PICO is its direct link with the 2×2 table of study results. The 2×2 table, or contingency table, is often used to present how often the outcomes (columns) occurred in the comparison groups (rows). The effect of the treatment on the risk of the outcome can then be calculated. Hence, the question and answer can be described very succinctly and intuitively in terms of a PICO and 2×2 table, respectively.

However, composing a PICO for diagnostic questions is less straightforward. The most commonly used PICO reflects questions about the accuracy of a medical test, but does not align with the 2×2 table for a test accuracy study. Also, the field of diagnostic test research has evolved to comprise patient important outcomes. The question is then which effect the use of a test has on patient health, and the conventional PICO and 2×2 table apply.

In this paper, I will illustrate how to make a PICO for both types of clinical questions about a medical test. First, I will present a PICO for a question about test accuracy that aligns with the 2×2 table of test accuracy studies. Next, I will show a PICO with a 2×2 table for a question about the effect of care with a test. The examples relate to breast cancer screening.

## Current convention

One PICO for questions about medical tests has been advocated by many: Population, Index test, Comparator test and test accuracy as Outcome.^2–4^ The index test is the new diagnostic or screening test under investigation, and the comparator test is the best available (reference) method for diagnosing the disease of interest. Although this PICO has strong face validity, it is ambiguous when studied in more detail. The index test as well as comparator test can be positive and negative and so it is unclear which results are compared. Additionally, the proposed outcome is not a health state but a statistical measure, such as sensitivity and specificity.

Moreover, the aim of some diagnostic research is to investigate the effect that offering a test has on patient health instead of test accuracy.^5^ This research acknowledges that the test result will determine which care the patient gets, and this will influence his health. It has been suggested that a PICO for a medical test should represent this type of research.^6 7^

Finally, although reviews about interventions are generally guided by an objective in terms of a PICO, reviews about test accuracy are not. Cochrane recommends to describe the objective of such a review as follows: ‘To determine the diagnostic accuracy of [index test] for detecting [target condition] in [participant description]’.^8^ This is obviously a clear and concise formulation, but the use of a PICO would promote consistency across reviews.

## PICO for test accuracy

Let’s say we want to know whether the result of a mammography (X-ray) reflects the presence of breast cancer accurately in women aged 40 years or older without symptoms. We could phrase this clinical question as follows: is the risk of having breast cancer higher in women with a positive mammography compared with women with a negative mammography? If so, how strong is the predictive strength of the test? The PICO for this and similar questions would be: P is the population of interest, I stands for positive index test result, C for negative index test result and O for the target disease (box 1).

Box 1 **PICO and 2×2 table for question about test accuracy**

Population of interest=women aged 40–74 years without a history of breast cancer

Investigated test result=positive result on mammography

Comparator test result=negative result on mammography

Outcome=breast cancer according to biopsy (or not)

**Table:** 

Group	Outcome	
	Breast cancer	No breast cancer
Positive index test	413 (a)	3026 (b)
Negative index test	12 (c)	65 315 (d)

Sensitivity=a/(a+c)=97.2%

Specificity=d**/**(b+d)=95.6%

Positive predictive value=a/(a+b)=12.0%

Negative predictive value=d/(c+d)=99.98%

Diagnostic OR=(a/b)/(c/d)=742.9

We would need to perform a test accuracy study to answer our question. The participants would get a mammography (index test) and a biopsy to determine the presence of breast cancer (reference test). Each test would yield a positive or negative test result. Preferably, all patients get both tests, without much time in between, and without the test interpreters knowing the result of the other test. However, to avoid subjecting a large group of women to an invasive biopsy, only women with a positive mammography get a biopsy. Cases in women with a negative mammography are detected as part of usual care, which includes a biopsy during 1 year of follow-up after the screening.

After finishing the study, we could present how many patients had breast cancer according to the biopsy (in the columns) by mammography result (in the rows). The figures in box 1 stem from the baseline measurement of the Swedish Two-County trial.^9 10^ Sensitivity and specificity were high (>95%), but since the prevalence of breast cancer was low (0.6%), the positive predictive value was very low (12.0%) and the negative predictive value very high (99.97%). In other words, the answer to our diagnostic question is that mammography picked up most cases of breast cancer but produced many false-positive results too.

## PICO for care with test

What we want to know next is whether screening with mammography improves women’s health. Are they better off if we provide this type of care? The outcome all-cause death would capture the targeted benefit (preventing death due to breast cancer) and possible fatal adverse events (due to repeated radiation or overdiagnosis leading to unnecessary treatment for breast cancer). If we set up a study, we would compare care with screening to care without screening. Hence, the PICO is composed in the same way as a PICO for a question about any other type of intervention (box 2).

Box 2 **PICO and 2×2 table for question about the effect of care with a test**

Population of interest=women aged 40–74 years without a history of breast cancer

Investigated intervention=care with breast cancer screening for 10 years

Comparator intervention=care without breast cancer screening for 10 years

Outcome=death (or not)

**Table:** 

Groups	Outcome	
	Deceased	Alive
Care with 10-year screening	7261 (a)	69 819 (b)
Care without 10-year screening	5252 (c)	50 733 (d)

OR=(a/b)/(c/d)=1.00

Relative risk=(a/(a+b))/(c/(c+d))=1.00

Absolute risk difference=(a/(a+b))−(c/(c+d))=+0.04%

We would need to perform an intervention study, preferably a randomised trial. Women aged 40 years or older without symptoms would be randomised to care with mammography or to usual care without mammography, and deaths in both groups monitored. As the yearly breast cancer incidence is low (<1%), a large number of women and long follow-up that spans many years would be needed to accrue sufficient events and so adequate power.

We could use a 2×2 table to present how many women died (in the columns) by intervention group (in the rows). The numbers in the box 2 are based on the Swedish Two-County trial that tested the effect of screening.^11^ Of the 77 080 women screened every 2–3 years during 10 years, 7261 died (9.4% in total: 0.2% from breast cancer, 9.2% from other disease). Among the 55 985 women who were not screened in that same period, 5252 died (9.4% in total: 0.3% from breast cancer, 9.3% from other disease). Hence, the answer to our clinical question is that women did not benefit from mammographic screening in terms of the risk of dying.

## Final remarks

In this paper, I distinguished two types of clinical questions about a medical test: is the test accurate, and does the use of the test improve patient health effectively? When teaching evidence-based medicine, it is important to make the distinction for several reasons. First, the nature of the two types of questions differs. A clinical question about a test’s accuracy relates to the accurate measurement of a health outcome: does the test result indicate the presence of the target disease good enough for a doctor to rely on it? In contrast, a question about an intervention based on such a test, such as, a screening programme, is about the intervention's effect on health: does the intervention change the risk of a certain health outcome in the participants?

Second, the type of question determines how the PICO is formulated. I explained the options above. Alternatively, some teachers might prefer to use just the one PICO structure that the two PICOs have in common on a meta-level: P stands for the population of interest, I for the investigated condition, C for the comparison condition and O for a health outcome. The choice is up to the teacher.

Third, the type of question determines which type of study answers the question. A test accuracy study compares the presence of the target disease in patients with a positive versus negative result on the index test cross-sectionally. An intervention study compares the health of patients who received care including the test with patients who did not receive this care during a certain period of follow-up. It could be an observational or randomised study.

Finally, the distinction between the two diagnostic questions is important, because high test accuracy does not automatically imply a health benefit to patients undergoing the test. The results of Swedish Two-County trial that I cited in the examples illustrated this point, and these findings have been corroborated by many other studies about breast cancer screening.^12–15^ Nevertheless, most diagnostic studies are designed to evaluate the test accuracy of a single test. More studies are needed that investigate the test accuracy of the customised diagnostic pathway with the new test, that is, the test strategy, or the effect that care based on this pathway has on health.^6 16^

## References

[1] Richardson WS , Wilson MC , Nishikawa J , et al . The well-built clinical question: a key to evidence-based decisions. ACP J Club 1995;123:A12–13. 10.7326/ACPJC-1995-123-3-A12 7582737

[2] Hawkins RC . The evidence based medicine approach to diagnostic testing: practicalities and limitations. Clin Biochem Rev 2005;26:7–18. 16278748 PMC1252824

[3] Scholten R , Offringa M , Assendelft WJJ . Inleiding in evidence-based medicine Klinisch handelen gebaseerd OP bewijsmateriaal. 5th edn. Houten: Bohn Stafleu van Loghum, 2018.

[4] Straus SE , Glasziou P , Richardson WS , et al . Evidence-Based medicine; how to practice and teach EBM. 5^th^ edition. Philadelphia: Elsevier, 2019.

[5] Knottnerus JA , van Weel C , Muris JWM . Evaluation of diagnostic procedures. BMJ 2002;324:477–80. 10.1136/bmj.324.7335.477 11859054 PMC1122397

[6] Ferrante di Ruffano L , Hyde CJ , McCaffery KJ , et al . Assessing the value of diagnostic tests: a framework for designing and evaluating trials. BMJ 2012;344:e686. 10.1136/bmj.e686 22354600

[7] Leeflang MMG , Deeks JJ , Takwoingi Y , et al . Cochrane diagnostic test accuracy reviews. Syst Rev 2013;2:82. 10.1186/2046-4053-2-82 24099098 PMC3851548

[8] Deeks JJ , Wisniewski S , Davenport C . Chapter 4: Guide to the contents of a Cochrane Diagnostic Test Accuracy Protocol. In: Deeks JJ , Bossuyt PM , Gatsonis C , eds. Cochrane Handbook for systematic reviews of diagnostic test accuracy version 1.0.0. The Cochrane collaboration, 2013. http://srdta.cochrane.org/

[9] Tabar L , Fagerberg CJG , Day NE . The results of periodic one-view mammography screening in a randomized controlled trial in Sweden. II Evaluation of the results. In: Day NE , Miller AB , eds. Screening for breast cancer. Toronto: Hans Huber Publishers, 1988: 39–44.

[10] Tabar L , Fagerberg CJG , South MC . The Swedish two-county trial of mammographic screening for breast cancer: recent results on mortality and tumour characteristics. In: Miller AB , Chamberlain J , Day NE , eds. Cancer screening. Cambridge: Cambridge University Press, 1991.

[11] Tabar L , Fagerberg G , Duffy SW , et al . The Swedish two County trial of mammographic screening for breast cancer: recent results and calculation of benefit. J Epidemiol Community Health 1989;43:107–14. 10.1136/jech.43.2.107 2512366 PMC1052811

[12] Mushlin AI , Kouides RW , Shapiro DE . Estimating the accuracy of screening mammography: a meta-analysis. Am J Prev Med 1998;14:143–53. 10.1016/s0749-3797(97)00019-6 9631167

[13] Gøtzsche PC , Jørgensen KJ , Cochrane Breast Cancer Group . Screening for breast cancer with mammography. Cochrane Database Syst Rev 2013;156. 10.1002/14651858.CD001877.pub5 PMC6464778 23737396

[14] Mandrik O , Zielonke N , Meheus F , et al . Systematic reviews as a 'lens of evidence': Determinants of benefits and harms of breast cancer screening. Int J Cancer 2019;145:994–1006. 10.1002/ijc.32211 30762235 PMC6619055

[15] Van Ourti T , O'Donnell O , Koç H , et al . Effect of screening mammography on breast cancer mortality: quasi-experimental evidence from rollout of the Dutch population-based program with 17-year follow-up of a cohort. Int J Cancer 2020;146:2201–8. 10.1002/ijc.32584 31330046 PMC7065105

[16] Bossuyt PM , Irwig L , Craig J , et al . Comparative accuracy: assessing new tests against existing diagnostic pathways. BMJ 2006;332:1089–92. 10.1136/bmj.332.7549.1089 16675820 PMC1458557

